# Characterization of the complete mitochondrial genome of *Tripetaloceroides tonkinensis* (Orthoptera: Tetrigoidea) from China and its phylogenetic analysis

**DOI:** 10.1080/23802359.2021.1938724

**Published:** 2021-06-14

**Authors:** Rong-Jiao Zhang, Lei Xin, Wei-An Deng

**Affiliations:** a School of Chemistry and Bioengineering, Hechi University, Yizhou, PR China; b Ministry of Education, Key Laboratory of Ecology of Rare and Endangered Species and Environmental Protection (Guangxi Normal University), Guilin, PR China; c Guangxi Key Laboratory of Rare and Endangered Animal Ecology, Guangxi Normal University, Guilin, PR China; d College of Life Sciences, Guangxi Normal University, Guilin, PR China

**Keywords:** Tetrigidae, Tripetalocerinae, *Tripetaloceroides tonkinensis*, mitogenome, phylogenetic analysis

## Abstract

The mitochondrial genome (mitogenome) of the *Tripetaloceroides tonkinensis* (Orthoptera: Tetrigoidea) was sequenced and annotated. The complete mitogenome has a length of 16,696 bp and consists of 13 protein-coding genes, 2 ribosomal RNA genes, 22 transfer RNA genes, and a A + T-rich region. Thirteen PCGs started with typical ATN codon and ended with complete stop codons (five with TAG, eight with TAA). The overall nucleotide composition was 42.7% of A, 10.34% of G, 25.87% of T, and 21.08% of C. The phylogenetic trees in the current study confirmed that *T*. *tonkinensis* was clustered with other Tetrigoidea species, and the study would improve our understanding for the mitogenomes of Tetrigoidea.

*Tripetaloceroides tonkinensis* (Günther 1938), belongs to the genus *Tripetaloceroides* Storozhenko, within the subfamily Tripetalocerinae of Orthoptera. This subfamily currently includes four known genera worldwide, which are mainly distributed in Southeast Asia (Storozhenko 2013), and there are one known genus and species in China (Deng 2016). However, up to now, no mitochondrial sequence has been reported for Tripetalocerinae (NCBI, last visited on April 2021). To further advance evolutionary studies for Tripetalocerinae, we sequenced and analyzed the mitochondrial genome of *Tripetaloceroides tonkinensis* (GenBank accession no. MW770353), which will help to better understand the phylogenetic status of this species in Tetrigoidea.

Total genomic DNA was extracted from legs of adult specimen of *T*. *tonkinensis* using the DNeasy Blood & Tissue Kit (Qiagen, Dusseldorf, Germany) according to the manufacturer’s instructions. The samples of *T*. *tonkinensis* were collected from Nonggang Nature Reserve in Guangxi province of China (22.474261°N, 106.957389°E) in May 2020 and voucher specimen is deposited in Entomological Museum of Hechi University, Yizhou, China (EMHU) (the voucher no. ii6). The genomic DNA was sequenced using the illumina Novaseq platform (Personalbio, Shanghai, China). The mitogenome was assembled using Geneious 10.2.3 (Kearse et al. 2012), and all genes were annotated with MITOS Web Server (Bernt et al. 2013).

The size of the mitogenome sequence obtained from *T*. *tonkinensis* was 16,696 bp. The gene composition, order, and orientation of *T*. *tonkinensis* was the same as the mitogenomes of other tetrigid species, and each sequence included 13 protein-coding genes(PCGs), 2 ribosomal RNA genes (*rrnL* and *rrnS*), 22 transfer RNA genes (*tRNAs*), and a A + T-rich region. The composition of the genome contained 42.7% A, 10.34% G, 25.87% T, and 21.08% C, showing an obvious A + T bias (68.57%). Nine PCGs and 14 *tRNAs* were transcribed from the majority strand, while the remaining four PCGs (*ND1*, *ND4*, *ND4L,* and *ND5*), eight *tRNAs*, and two *rRNAs* were located on the minority strand. Thirteen PCGs started with typical ATN codon (one with ATC, three with ATA, four with ATT, and five with ATG) and ended with complete stop codons (five with TAG and eight with TAA), which were presumably completed as TAA by post transcriptional polyadenylation (Anderson et al. 1981). A total of 22 *tRNAs* were found interspersed in the mitogenomes of *T*. *tonkinensis*, which ranged in size from 62 bp (*trnD* and *trnF*) to 69 bp (*trnQ* and *trnM*). The two ribosomal RNA genes (*rrnL* and *rrnS*) occurred in *T*. *tonkinensis* mitogenomes between *trnL1* and the A + T-rich region, separated by *trnV* gene. The lengths of *rrnS* and *rrnL* determined in *T*. *tonkinensis* were 737 bp and 1230 bp, respectively.

The phylogenetic relationships of *T*. *tonkinensis* were reconstructed using Bayesian Inference (BI) by MrBayes 3.2.6 (Ronquist et al. 2012), i.e. the BI tree was produced (Figure 1) based on 13 PCGs (10,911 bp) from mitogenomes of 15 tetrigid species and one outgroup (*Myrmecophilus manni*), respectively. As shown in Figure 1, Tetrigoidea was retrieved as monophyletic with strong support (posterior probability, PP = 1). *T. tonkinensis* split off earliest from the other taxa, was positioned as a sister group to the remaining Tetrigoidea (PP = 1), suggesting that it is the earliest species within Tetrigoidea.

**Figure 1.**
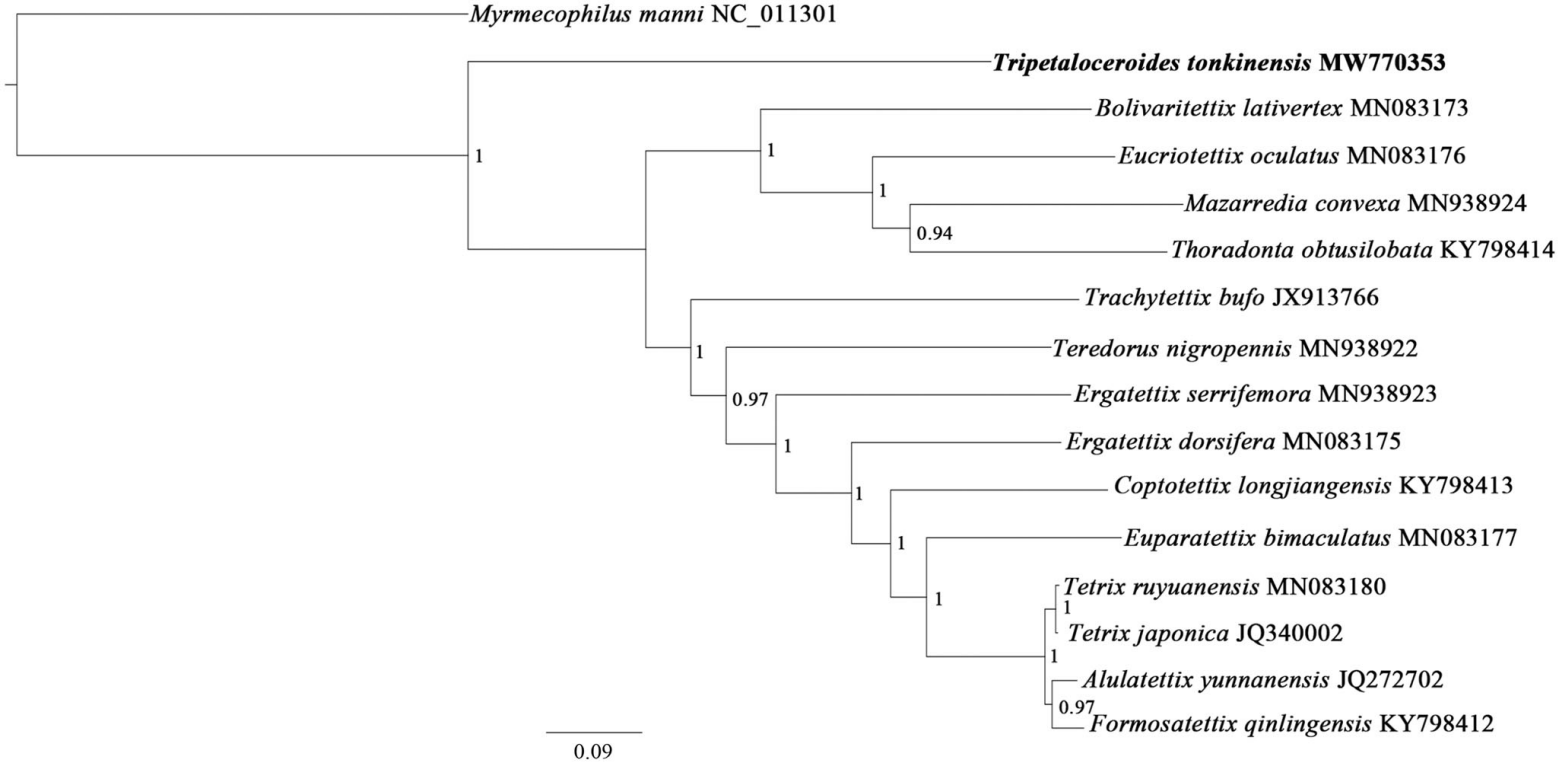
Phylogenetic tree obtained from BI analysis based on 13 concatenated mitochondrial PCGs. Values at nodes indicate BI posterior probabilities (PP).

## Data Availability

The data that support the findings of this study are openly available in National Center for Biotechnology Information at https://www.ncbi.nlm.nih.gov/nuccore, Reference no. MW770353.
